# The effects of music on cardiorespiratory endurance and muscular fitness in recreationally active individuals: a narrative review

**DOI:** 10.7717/peerj.13332

**Published:** 2022-04-22

**Authors:** Francesca Greco, Elisa Grazioli, Loretta Francesca Cosco, Attilio Parisi, Maurizio Bertollo, Gian Pietro Emerenziani

**Affiliations:** 1 Department of Experimental and Clinical Medicine, University of Catanzaro “Magna Græcia”, Catanzaro, Italy; 2 Department of Human Movement Sciences and Health, University of Rome “Foro Italico”, Rome, Italy; 3 Behavioral Imaging and Neural Dynamics Center, Department of Medicine and Aging Sciences, University “G. d’Annunzio” of Chieti-Pescara, Chieti, Italy

**Keywords:** Aerobic exercise, Music, Strength training, Physical fitness

## Abstract

Music is made up of several features (*e.g*., melody and rhythm) and it accompanies our life in different daily activities. During the last years, there was a growing interest in research about the music-related effects in the exercise domain. Music stimuli could act as an ergogenic effect leading to improvements in health-related and physical fitness components like cardiorespiratory endurance and muscular fitness. Moreover, listening to music may positively affect individuals’ psychological state which could lead to increased exercise adherence. Conflicting data exist regarding the effects of music on cardiorespiratory and muscle-strengthening exercises indicating that music’s characteristics (*i.e*., rhythm and musicality), studied samples (*i.e*., athletes and amateur) and methodology (*i.e*., self-selected music and research-selected music) might influence the results. Listening to music while exercising is becoming more frequent also in recreationally active individuals. While literature mainly focused on the effects of music in elite and amateur athletes, little data are available regarding recreationally active participants. Therefore, this review aims to summarize evidence regarding the effects of music on health-related physical fitness components in recreationally active individuals, specifically referring to cardiorespiratory endurance and muscular fitness. These outcomes will be helpful to all recreationally active participants to optimize the exercise protocol with the use of music.

## Introduction

Music has been a part of human culture throughout history, and it has been linked to health and emotional well-being for thousands of years (Karageorghis, 2020). Music has different fundamental features (*i.e*., rhythm, melody, harmony, and dynamics) and since it is widely varied, it could be classified in multiple ways (*e.g*., by music genre, date of release, musical tempo). According to the inaugural conceptual framework to predict the effects of music in sport and exercise developed by Karageorghis and colleagues, properties of the music itself (*i.e*., rhythm, musicality) and external factors (*i.e*., association and cultural influence) affect the motivational qualities of music, which should act as ergogenic aid able to enhance the physiological and psychological status of participants during sport-related activities and physical exercise (PE) (Karageorghis, Terry & Lane, 1999; Karageorghis & Priest, 2012a). Through the manuscript, the term *“physical exercise” (PE)* will be used to indicate a planned and structured subcategory of physical activity (PA), which aims to maintain or enhance *physical fitness* (PF). Health-related components, such as cardiorespiratory endurance, body composition, muscular fitness, and flexibility, are part of PF (Caspersen, Powell & Christenson, 1985). More recently, Karageorghis (2016) updated his original conceptual framework, including personal factors (*e.g*., musical preference, training status) and situational variables (*i.e*., context, task). These factors could influence enjoyment, which is a predictor of adherence to PE, maximizing health-related benefits, and playing a motivational role in exercising (Rhodes & Kates, 2015; Terry et al., 2020). Moreover, several studies have been conducted on the music tempo (*e.g*., 120 bpm) as a dominant characteristic to consider while exercising, with specific attention given to the use of music in the sport context or as a therapeutical approach (*i.e*., music therapy) (Terry et al., 2020; Bonavolontà et al., 2021; Thaut, 2015). The application of music before, during, and after cardiorespiratory exercise has been the aim of extensive research literature in athletes (Terry et al., 2020; Karageorghis, 2017; Carlier et al., 2017; Dyrlund & Wininger, 2008), despite its capacity to influence affective responses positively, pleasure and enjoyment during continuous exercise are not well documented in active people.

Therefore, studies on the effects of listening to music on health-related parameters are needed in recreationally active individuals. Due to its ergogenic and pleasant effect, music stimuli could represent a motivational support and then a protective factor to prevent physical inactivity and sedentarily.

The positive effects of the regular practice of PA on health-related outcomes are widely recognized in all age groups (World Health Organization, 2020). Physical activity plays a crucial role in primary and secondary prevention of more than 25 chronic diseases, such as cardiovascular, metabolic, and cancer disease, as well as on premature mortality (Grazioli et al., 2017; Warburton, Nicol & Bredin, 2006; Pedersen & Saltin, 2015). Indeed, cardiorespiratory and muscle-strengthening exercises positively influence several aspects of health status (*e.g*., cardiovascular and respiratory functioning, body composition). Myers et al. (2015) and colleagues have reported a risk reduction for premature mortality (10–25%) for every 1-metabolic equivalent (MET) increase in aerobic fitness both in men and women, even in those with lower aerobic capacities (*i.e*., <5 METs). Moreover, muscle-strengthening training can prevent several disabling symptoms related to ageing, such as risks of fall, sarcopenia, dynapenia, loss of independence, risk of disability, and mortality (Garber et al., 2011; Bennie, Shakespear-Druery & De Cocker, 2020). Current international guidelines suggest minimizing the time spent in sedentary behaviour exercising at least 150– 300 min of moderate-intensity aerobic PA or at least 75–150 min of vigorous-intensity aerobic PA. Moreover, strength training should be done two or more days per week at a moderate or greater intensity that involves all major muscle groups (World Health Organization, 2020). Despite the well-recognized benefits of PA and the side effect of sedentary behaviour, millions of people are physically inactive and more alarming, the recent reports evidenced that the prevalence of physical inactivity is growing (Mclaughlin et al., 2020). It seems that several barriers influenced the low adherence in regular PA, such as lack of time and a low motivation (Reichert et al., 2007) and issues related to volition. According to this evidence, it seems urgent to find new strategies able to increase PA compliance. In this context, music, an inexpensive, accessible, and artistic stimulus, might be an effective way to motivate exercise participation.

Previous reviews considered the role of music independent of the type of exercise proposed or mainly focused on the effects of pre-task music among sports competitors (Karageorghis & Priest, 2012a; Terry et al., 2020; Karageorghis & Priest, 2012b; Smirmaul, 2017). Other reviews evidenced the effects of music in a cardiac rehabilitation setting (Chair, Zou & Cao, 2021), targeting a clinical and older adult population (Clark, Taylor & Baker, 2012; Ziv & Lidor, 2011) or considering the influence of the preferred music (Ballmann, 2021). Moreover, in the existing reviews, there is no uniformity regarding the descriptive characteristics of the population of interest and are not focused only on recreationally active participants (meet World Health Organization minimum activity guidelines and may participate in multiple sports/forms of activity) as classified by McKay et al. (2022) as Tier 1.

Due to the availability of technological devices at low prices, recreationally active individuals have been also started to listen to music while exercising. As the effects of music in elite and amateur athletes are better known (Smirmaul, 2017; Terry et al., 2020), few data are available regarding recreationally active participants. Therefore, the current review aimed to provide evidence on the effects of music on health-related physical fitness components, specifically referring to cardiorespiratory endurance and muscular fitness in recreationally active individuals.

## Survey methodology

A literature search was conducted using three databases (PubMed, Scopus and Web of Science) for the present narrative review. The search was limited to peer-reviewed journals written in the English language. Keywords used in searches included “music”, “physical activity”, “high-intensity training”, “aerobic performance”, “endurance performance”, “exercise”, “endurance”, “strength”, and “resistance” with no time limits. Inclusion criteria consisted of studies regarding the effects of different types of music on exercise performance in recreationally active participants. The term “recreationally active” refers to healthy and physically active individuals not involved in some forms of organized sports (*i.e*., amateur athletes), belonging to Tier 1 according to Participant Classification Framework (McKay et al., 2022). Articles were excluded if they focused on clinical practice (*i.e*., music therapy) and élite athletes (*i.e*., competing at the national level or with a purpose to compete). A manual search of the reference lists in the studies found in the computerized search was also conducted. Once the duplicates were removed, the comprehensive search yielded 22 studies that matched the criteria mentioned above. A summary of the reviewed studies is presented in Table 1.

**Table 1:** Music in cardiorespiratory endurance and muscle-strengthening exercises: study’s characteristics.

Study	Participants	Music conditions	Activity	Primary findings
Archana & Mukilan (2016)	18 males (22.45 ± 1.86 years) 12 females (22.81 ± 1.72 years)	NM; M	15 min of moderate aerobic exercise on bicycle ergometer	Lower HR during exercise in Music than NM
Cole & Maeda (2015)	20 women and 15 men undergraduate active students (20.7 ± 2.3 years)	NM, NON-PREF; PREF	12 min Cooper Test	Higher running performance in female listening to PREF
Dyrlund & Wininger (2008)	200 healthy people (126 females, 74 males - 20.7 ± 4.4 years)	NM; NON-PREF (130 bpm); PREF (130 bpm)	20 min activity at low (30% VO_2 max_), or moderate (50% VO_2_ max), or high (70% VO_2__max_)	PREF small effect on exercise enjoyment
Ghaderi, Rahimi & Azarbayjani (2009)	30 male physical education college students (25.66 ± 3.89 years)	MOT; REL; NM	Treadmill running to exhaustion (80-85% of maximal HR)	Greatest aerobic performance with MOT; Lowest RPE and salivary cortisol levels with REL
Jones, Tiller & Karageorghis (2017)	13 males runners (20.2 ± 1.9 years)	ST (55–65 bpm); FT (125–135 bpm); NM	Three exercise sessions of high-intensity intervals interspersed with a 3–10 min passive recovery period	Higher feeling Scale scores throughout recovery periods with FT
Köse & Atli (2019)	35 Sport Science male students (22.63 ± 2.9 years)	ST (100 bpm); FT (140 bpm); NM	Bruce treadmill test with 72-h intervals	Greatest performance with FT; Lowest lactate concentration after recovery with ST
Patania et al. (2020)	19 physically active people (26.4 ± 2 years)	NM; LOW (90–110 bpm), MED (130–150 bpm), HIGH (170–190 bpm)	walking for 10 min at 6.5 km/h on a treadmill or Leg Press at 80% on 1-RM	Lower RPE during walking in LOW, MED and HIG groups *vs* NM; in MED and HIGH *vs* LOW. Lower RPE after Leg Press in MED and HIG *vs* NM groups
Arazi, Asadi & Purabed (2015)	12 well-trained resistance exercise males (24 ± 2 years)	WU+RE with music (130 bpm); WU+RE without music; WU with music+RE; WU without music+RE	Resistance exercises circuit	Higher RPE, HR, blood pressure in WU+RE without music condition
Ballmann et al. (2020)	10 resistance-trained males (21.6 ± 1.7 years)	PREF (>120 bpm); NON-PREF	Bench press resistance exercise (at 75% of 1-RM)	Greatest motivation and repetitions to failure with PREF
Ballmann et al. (2021)	12 resistance-trained college-aged males (20.5 ± 1.24 years)	PREF (127 ± 28 bpm); NON-PREF (126 ± 25 bpm)	Resistance exercise (bench press at 75% 1-RM)	Greatest repetitions, mean velocity, relative mean power, peak velocity, peak power and motivation with PREF
Bartolomei, Di Michele & Merni (2015)	31 resistance-trained men (26.6 ± 6.8 years)	SSM (>120 bpm); NM	Maximal bench strength (1-RM) and endurance (repetitions to failure at 60% 1-RM)	Greatest endurance performance with SSM
Biagini et al. (2012)	20 resistance-trained men (22.95 ±1.90 years)	SSM; NM	Bench press strength-endurance (3 sets at 75% 1-RM) and squat jump (3 reps at 30% back squat 1-RM)	Greatest take-off velocity, rate of velocity development, and rate of force development with SSM in squat jump exercise; lower RPE with SSM in squat jump exercise; greatest fatigue, vigour and tension with SSM
Bigliassi et al. (2018)	19 healthy adults (24.2 ± 4.9 years)	Music track (119 bpm); NM	Hand grip test	Greatest dissociative attention and upregulated affective arousal with music
Crust (2004)	27 undergraduates sport science males (20.2 ± 1.7 years)	SSM (>120 bpm); white noise	Isometric weight-holding	Greatest endurance performance with SSM
Cutrufello, Benson & Landram (2020)	8 men and 7 women healthy college-aged students (20.1 ± 1.79 years)	SSM; NM	Bench press endurance (5 sets at 70% 1-RM)	Greatest endurance performance with SSM
Feiss et al. (2021)	63 strength-trained young adults (25.0 ± 4.4 years)	FT (120 bpm); ST (90 bpm); NM	Wall-sit and plank-hold exercises	Longer dissociative state during wall-sit exercise with both FT and ST
Karageorghis, Drew & Terry (1996)	25 men (22.9 ± 2.8 years) and 25 women (24.0 ± 3.8 years) volunteer sport science undergraduates	STIM (134 bpm); SED (90 bpm); white noise	Hand grip test	Higher grip strength with STIM
Köse (2018)	26 male students (23.92 ± 2.05 years)	SSM (>120 bpm); NM	Maximal bench press (1RM) and endurance (60% of 1RM)	Greatest endurance performance with SSM
Moss, Enright & Cushman (2018)	16 resistance-trained males (22.0 ± 3.4 years)	SSM (129 ± 9 bpm); electronic dance music (128 ± 1 bpm); metal (159 ± 24 bpm); NM	Power based (30% 1-RM) and strength-based (60%, 70% and 80% 1-RM) repetition to failure exercise protocol in the bench press and back squat exercises	Repetitions to failure increased by a small to moderate amount for all music conditions at low but not high intensities; increased vigour in all music conditions
Pearce (1981)	Undergraduate students (33 males and 16 females)	STIM; SED; NM	Hand grip test	Reduced grip strength with SED
Silva et al. (2021)	20 young undergraduate students (20.0 ± 1.4 years)	PMG; NPM; NM	Hand grip test; lat-pulldown strength-endurance (75% of 1-RM)	Greatest grip strength and endurance performance with PMG; Greatest RPE with NM
van den Elzen et al. (2019)	153 healthy community dwelling people (73.0 ± 6 years)	PREF; most disliked music; NM	Hand grip test	Greatest grip strength with PREF

**Note:**

FT, fast-tempo; HR, heart rate; M, music according to the International Organization For Standardization MOT, motivational music; NM, no music; NON-PREF, non-preferred music; NPMG, non-preferred music genre; PMG, preferred music genre; PREF, preferred music; RE, resistance exercise; REL, relaxation music; RPE, rate of perceived exertion; STIM, stimulative music; SED, sedative music; SSM, self-selected music; ST, slow-tempo; WU, warm-up.

### The role of music on cardiorespiratory endurance in recreationally active individuals

The use of music as a work-enhancing effect is mainly derived by studies in literature using aerobic exercises models (*e.g*., running, cycling, walking). Several studies have focused on the efficacy of music on aerobic performance (Karageorghis & Terry, 1997; Urakawa & Yokoyama, 2005) and the rating of perceived exertion (RPE) (Boutcher & Trenske, 1990; Potteiger, Schroeder & Goff, 2000). Therefore, this section highlights the main findings on the application of music in cardiorespiratory tasks, also considering pleasure and enjoyment in recreationally active exercisers.

Regarding high-intensity exercise (HIIT), few studies investigated the influence of music on adherence, performance and motivation (Thum et al., 2017; Jones, Tiller & Karageorghis, 2017). HIIT is one of the most used protocols nowadays which requiring less time than conventional training. However, the shortness of breath, leg pain, and fatigue usually experienced during HIIT may make this modality less tolerable in certain populations (Thum et al., 2017). The latest research focused on the effects of music during HIIT demonstrating that music could mitigate the decline in pleasure typically experienced during this protocol and seemed to increase enjoyment in postexercise (Thum et al., 2017). Listening to music on psychophysiological variables was also investigated during the recovery period after HIIT exercise (Jones, Tiller & Karageorghis, 2017). Specifically, participants performed three bouts of HIIT with 3 min of passive recovery between each bout. Three different recovery phase conditions were proposed to the individuals: positively-valenced music of a slow-tempo (55–65 bpm), fast-tempo (125–135 bpm), and no-music. Results showed that fast-tempo music applied during recovery periods produced a more pleasant experience, evaluated with Feeling Scale score, but no differences between groups were found in RPE and cardiorespiratory indices (Jones, Tiller & Karageorghis, 2017). Although these results seem promising, other studies are needed to investigate better the effects of music on cardiorespiratory endurance, RPE and adherence in different populations. The latter mentioned studies (Thum et al., 2017; Jones, Tiller & Karageorghis, 2017) implemented the evidence reported by Dyrlund & Wininger (2008) that investigated the effects of intensity and music on psychological variables. Participants walked or ran on a treadmill for 20 min under different conditions: three music conditions (most preferred music, least preferred music, or no music) and three exercise intensity (low, moderate, or high). Data showed a strong influence of exercise intensity on RPE and attention, while the most preferred music produced a small effect on exercise enjoyment, probably due to a bit of increased participants’ attention. No differences were found on the RPE during the three music conditions (Dyrlund & Wininger, 2008).

In contrast to the few studies focused on the effects of music on HIIT, an extensive number of studies focused on maximal and submaximal exercise tests (Urakawa & Yokoyama, 2005; Potteiger, Schroeder & Goff, 2000; Cole & Maeda, 2015; Köse & Atli, 2019). Ghaderi, Rahimi & Azarbayjani (2009) investigated during submaximal endurance tests the effect of relaxational and motivational music on aerobic performance, RPE, and the salivary cortisol. Thirty physical education college students had to run to exhaustion during submaximal running at the intensity of 80–85% of their maximal heart rate (HR) on a treadmill under three different conditions (motivational music, relaxation music, and no-music). The main results showed the highest aerobic performance in the motivational music group; the relaxation group had significantly lower RPE values than the other two groups (Ghaderi, Rahimi & Azarbayjani, 2009). Moreover, salivary cortisol concentrations were significantly lower at 5 min after exercise in relaxation music than motivational- and no- music conditions (Ghaderi, Rahimi & Azarbayjani, 2009). Cole and Maeda tried to understand if music could have a different impact on aerobic performance depending on gender (Cole & Maeda, 2015). They evaluated the effect of preferred, nonpreferred and no music on aerobic performance, through the 12-min Cooper Test in 20 women and 15 men who reported running at least once per week. Results showed that preferred music could improve running performance in women but not in men, suggesting a higher sensitivity to music by female subjects and a possible sex difference (Cole & Maeda, 2015). Other studies are needed to verify this hypothesis.

A study conducted on college students evidenced that also music tempos could influence aerobic performance and perceived fatigue (Köse & Atli, 2019). After a Bruce treadmill test, results reported that fast tempo music (140 bpm) improved running time compared to no music and slow tempo music (100 bpm) conditions. However, fast tempo did not affect on HR and RPE during and after exercise. Lastly, lactate concentration evaluated during 15 min of recovery phase was found to be lower with slow tempo music compared to fast tempo and no-music condition (Köse & Atli, 2019). This study highlights the potential impact of different tempo music on aerobic performance, suggesting the usage of a fast tempo music to motivate active individuals and to provide an ergogenic aid. In contrast, slow tempo music could be used to enhance the positive effects of recovery after exercise (Köse & Atli, 2019). These results are supported by Patania and colleagues who evidenced that music has a higher effect on RPE during endurance (walking for 100 at 6.5 km/h on a treadmill) than during high intensity (80% on 1-RM) exercise in active women (Patania et al., 2020). Participants were randomly assigned into four different conditions: no music, music at 90–110 bpm (LOW), music at 130–150 bpm (MED), and music at 170–190 bpm (HIGH). Results showed decrease RPE in the endurance group that listened to LOW, MED and HIG music compared to no music. Moreover, participants who listened to MED and HIGH music had a greater RPE reduction than the LOW group (Patania et al., 2020). Most of the studies, conducted on healthy college students, highlighted not only the effect of music on performance and fatigue, but also on the emotional state during training and performance (Karageorghis, Jones & Stuart, 2008; Bigliassi et al., 2013; Nakamura et al., 2010; Stork et al., 2015). Karageorghis and colleagues evidenced that interest-enjoyment was higher for medium tempi (115–120 bpm) than mixed tempi (Karageorghis, Jones & Stuart, 2008). The results from Bigliassi et al. (2013) showed that music acts efficiently as an ergogenic aid, particularly at submaximal exercise intensity, suggesting that, in the case of tasks around 70% of maximum aerobic capacity, beats around 115 to 125 could be more appropriated to elicit positive emotions. Lastly, Archana and colleagues suggested that listening to preferential music, during cycling activity could be an effective relaxation method (Archana & Mukilan, 2016). They analysed the base line ECG of 30 healthy male and female volunteers during a moderate exercise on a bicycle ergometer for 15 min, with and without music. Data showed an improvement in cardiac efficiency. In fact, the participants who listened to the music could exercise at lower HR than no music. Therefore, listening to preferred music along with exercise could have a calming effect on the mind (Archana & Mukilan, 2016).

### The role of music on muscular fitness in recreationally active individuals

Scientific evidence has shown different results regarding the effects of listening to music on muscle-strengthening exercises (*i.e*., resistance exercises). Most scientific literature has been focused on the effects of music on muscle-strengthening physical performance, psychological (*e.g*., mood, feeling status, enjoyment), psychophysical (*e.g*., RPE), and physiological (*e.g*., HR) responses in recreationally active participants (Biagini et al., 2012; Bartolomei, Di Michele & Merni, 2015; Ballmann et al., 2021; Silva et al., 2021; Moss, Enright & Cushman, 2018; Feiss et al., 2021). Therefore, this section highlights the main findings on the application of music in muscular fitness tasks in recreationally active exercisers.

One of the earliest studies involved undergraduates students (33 males and 16 females) showed that the types of music influenced the handgrip strength test differently (Pearce, 1981). Indeed, sedative music reduced grip strength relative to stimulative music and silence, otherwise stimulative music did not increase strength relative to no-music condition (Pearce, 1981). Shortly after, another study showed a higher grip strength after listening to upbeat music (134 bpm) than relaxing music (90 bpm) and white noise condition in 25 males and 25 females young adults (Karageorghis, Drew & Terry, 1996). Moreover, men had higher grip strength than women across all music conditions and both genders produced the highest force following stimulative music (Karageorghis, Drew & Terry, 1996). The effects of music during an isometric muscular endurance task revealed that undergraduate males students held a suspended weight for a significantly longer time while listening to self-selected music (SSM), characterized by a fast tempo >120 bpm, compared to a white noise condition (Crust, 2004). Moreover, preferred music increased handgrip performance compared to most disliked and no-music conditions in healthy older adults (van den Elzen et al., 2019).

The results from Biagini et al. (2012) showed that SSM had a worked-up effect on explosive strength and velocity (squat jump exercise performed at 30% back squat 1RM) but not in bench press strength-endurance (at 75% 1RM) in 20 young resistance-trained men. Moreover, participants indicated lower RPE and greater fatigue, vigor, and tension in the SSM condition than no music condition while performing squat jump exercise (Biagini et al., 2012). In contrast, Bartolomei, Di Michele & Merni (2015) showed positive effects of SSM (characterized by a fast rhythm >120 bpm) on bench press strength-endurance exercise (60% of 1RM) while no effects were found in maximal bench press strength (1RM) in 31 resistance-trained men. The results from Bartolomei, Di Michele & Merni (2015) were confirmed by another study which also found no improvements in 1RM bench press but an increase in strength-endurance (60% of 1RM) while listening to motivational music (>120 bpm) compared to a no-music condition (Köse, 2018). Cutrufello, Benson & Landram (2020) showed that SSM improved bench press performance (70% of 1RM) in healthy students compared to a no-music condition. The findings of Ballmann et al. (2021) suggested that listening to the preferred music rather than the nonpreferred one increases motivation and resistance performance (bench press exercise with loads at 75% 1RM) in 12 resistance-trained males. Moreover, Ballmann et al. (2020) examined the effects of listening to preferred music during warm-up revealing positive effects on bench press exercise (75% at 1RM) and motivation compared to nonpreferred music in young resistance-trained males. However, music did not affect barbell velocity and RPE (Ballmann et al., 2020).

Regarding the music genre choice, Moss and colleagues showed no additional benefits of electronic dance music, SSM, and metal on mean power output and mean velocity production during both squat jump and bench press exercise in 16 trained males (Moss, Enright & Cushman, 2018). Moreover, RPE was not significantly lower across conditions (Moss, Enright & Cushman, 2018). More recently, Silva and colleagues found that listening to the preferred music genre could increase maximal handgrip strength and upper body strength endurance (lat-pulldown exercise at 75% of 1RM) (Silva et al., 2021). Lower RPE was reported when listening to the preferred music compared to nonpreferred and no-music conditions during strength-endurance exercise in young adults (Silva et al., 2021). Instead, results from Feiss et al. (2021) revealed that, while performing wall-sit and plank exercises, the presence of music (fast and slow tempo: 120 and 90 bpm respectively) did not affect HR, RPE, or affective responses in 63 physically active young adults. However, both fast and slow-tempo music promoted a dissociative effect in the wall-sit exercise and overall, both music conditions were well-liked, as reported by the music enjoyment scale (Feiss et al., 2021). The use of music during warm-up and while performing a resistance circuit revealed lower levels of RPE, HR, and higher systolic blood pressure compared to a warm-up and resistance circuit done in the no-music condition in 12 strength-trained males (Arazi, Asadi & Purabed, 2015). The results from Bigliassi et al. (2018) found no significant differences in RPE while performing an isometric grip task in 19 healthy young adults although music increased dissociative attention.

## Conclusions

Music might act as an ergogenic aid in recreationally active participants during cardiorespiratory and muscle-strengthening exercises, especially in strength-endurance tasks. However, the heterogeneity of the findings does not lead to a conclusive result. Indeed, the different exercise intensities, research design and methods might have influenced the outcomes differently. Listening to music could also modulate the affective responses (*e.g*., feeling status, enjoyment) during exercise. Therefore, it should be taken into consideration when realizing training protocols. Among the music characteristics, the type of music (*e.g*., self-selected, motivational), plays a crucial role in modulating the effects on exercise performance. Indeed, motivational and self-selected music positively affects cardiorespiratory and muscular fitness components. Therefore, we suggest listening to self-select music tracks while exercising. Studies regarding the optimal aerobic and muscle-strengthening exercises strategies with music are strongly needed, especially in recreational exercisers. Moreover, it would be interesting to investigate the effects of music at different points of the training session (*e.g*., warm-up and cool-down), in different populations (*e.g*., inactive *vs* active individuals), and in longitudinal interventional studies.

## Additional Information and Declarations

### Data Availability

The following information was supplied regarding data availability: The article is a literature review.
